# Autochthonous Transmission of West Nile Virus by a New Vector in Iran, Vector-Host Interaction Modeling and Virulence Gene Determinants

**DOI:** 10.3390/v12121449

**Published:** 2020-12-16

**Authors:** Nariman Shahhosseini, Seyed Hassan Moosa-Kazemi, Mohammad Mehdi Sedaghat, Gary Wong, Sadegh Chinikar, Zahra Hajivand, Hamid Mokhayeri, Norbert Nowotny, Mohammad Hassan Kayedi

**Affiliations:** 1 Département de Microbiologie-Infectiologie et d’Immunologie, Université Laval, Québec City, QC G1V4G2, Canada; gary.wong@crchudequebec.ulaval.ca; 2 Department of Medical Entomology & Vector Control, School of Public Health, Tehran University of Medical Sciences, Tehran 141556446, Iran; shm.kazemi@gmail.com (S.H.M.-K.); sedaghat@hotmail.co.uk (M.M.S.); hajivand.zahra93@gmail.com (Z.H.); 3 Pasteur Institute of Shanghai, Chinese Academy of Sciences, Shanghai 200031, China; 4 Pasteur Institute of Tehran, Tehran 1316943551, Iran; sadeghchinikar@yahoo.com; 5 Department for Pathobiology, Institute of Virology, University of Veterinary Medicine, 1210 Vienna, Austria; norbert.nowotny@vetmeduni.ac.at; 6 Health Center, Department of Communicable Diseases Control and Prevention, Lorestan University of Medical Sciences, Khorramabad 6813833946, Iran; hmokhayeri@yahoo.com; 7 Department of Basic Medical Sciences, College of Medicine, Mohammed Bin Rashid University of Medicine and Health Sciences, Dubai 505055, UAE; 8 Razi Herbal Medicines Research Center and Department of Parasitology and Mycology, School of Medicine, Lorestan University of Medical Sciences, Khorramabad 6814993165, Iran

**Keywords:** West Nile virus, *Cx. theileri*, *Cx. pipiens*, phylogeny, virulence factors, Iran

## Abstract

Using molecular techniques and bioinformatics tools, we studied the vector-host interactions and the molecular epidemiology of West Nile virus (WNV) in western Iran. Mosquitoes were collected during 2017 and 2018. DNA typing assays were used to study vector-host interactions. Mosquitoes were screened by RT-PCR for the genomes of five virus families. WNV-positive samples were fully sequenced and evolutionary tree and molecular architecture were constructed by Geneious software and SWISS-MODEL workspace, respectively. A total of 5028 mosquito specimens were collected and identified. The most prevalent species was *Culex (Cx.) pipiens* complex (57.3%). Analysis of the blood-feeding preferences of blood-fed mosquitoes revealed six mammalian and one bird species as hosts. One mosquito pool containing non-blood-fed *Cx. theileri* and one blood-fed *Culex pipiens pipiens (Cpp.)* biotype *pipiens* were positive for WNV. A phylogram indicated that the obtained WNV sequences belonged to lineage 2, subclade 2 g. Several amino acid substitutions suspected as virulence markers were observed in the Iranian WNV strains. The three-dimensional structural homology model of the E-protein identified hot spot domains known to facilitate virus invasion and neurotropism. The recent detection of WNV lineage 2 in mosquitoes from several regions of Iran in consecutive years suggests that the virus is established in the country.

## 1. Introduction

Arthropods are a principal source of many emerging viruses in the human population. There is still much to learn about the interaction of arthropod-borne viruses (arboviruses) and their respective natural hosts, as well as the changes in virus-host ecology resulting in cross-species transmission events [1]. Arbovirus infections cause illness with serious health, welfare and socio-economic implications [2]. More specifically, mosquito-borne viruses constitute a major challenge for public and veterinary health, causing epidemics or epizootics [3,4,5]. Continuing eco-climatic change and globalization create suitable conditions for the emergence of mosquito-borne viruses in previously naive areas [5,6], including Iran [7,8]. The first detection of West Nile virus (WNV) in Iran dates back to 2009, when an Iranian patient presented with encephalitis died in Central Iran [9].

WNV belongs to family *Flaviviridae*, genus *Flavivirus* and is a member of the Japanese encephalitis (JE) virus serocomplex [10]. WNV can be transmitted by several mosquito species, primarily from the *Culex* genus but can also be transmitted through organ transplantation, blood transfusion and trans-ovarian routes [11]. Birds are amplifying hosts in the transmission cycle of WNV, while mosquitoes can acquire infection by feeding on viremic birds and act as a vector. Although the infection in many bird species is asymptomatic, severe disease resulting in death can be observed in some bird species such as crows, jays and birds of prey [12]. Humans and horses are considered dead-end hosts. Infections in humans are generally asymptomatic but about 20% of cases develop influenza-like symptoms (WNV fever), while 1% of cases develop neuroinvasive disease leading to death [13,14].

Phylogenetic analyses suggested up to nine genetic lineages of WNV [15]. Lineage 1 is widely distributed and further divided into lineage 1a, which includes the American strains [10,14]; lineage 1b, which is also referred to as Kunjin virus and mainly described in Australia; and lineage 1c, which is also referred to as lineage 5 and comprises isolates from India [16]. Lineage 2 was restricted to Sub-Saharan Africa until its emergence in Europe around 2004 [6] and spread across Europe since 2008 [17], lineage 3 (Rabensburg virus) has only been isolated in the Czech Republic [18] and lineage 4 was detected in 1998 in *Dermacentor* ticks in the Causcasus/Russia (more recently described as lineage 4a) [19]. A putative sixth lineage has been reported from Spain [20], which is now considered lineage 4b. A Malaysian Kunjin virus strain, which is genetically different to the Australian Kunjin strains, is now listed as lineage 5 [21]. Putative lineages 6 (Koutango virus) and 7 have been described from Senegal (for the latter there is only a partial sequence available, thus it is not included in the phylogenetic tree) [22]. Lineage 9, or, more appropriately, lineage 4c, was recently identified in *Uranotaenia unguiculata* mosquitoes in Austria [23].

WNV lineages 1 and 2 are known as pathogenic strains for humans. Nonetheless there are virus strain-specific differences in pathogenicity. For example, in lineage 2, it has been demonstrated that some South African WNV strains (e.g., SPU116/89, SA93/01, H442) are virulent with a neuroinvasive phenotype, in contrast, some other lineage 2 strains are less neuroinvasive, such as WNV strains in Madagascar (AnMg798, 1978), Cyprus (CYP68) and South Africa (SA381/00) [24]. The differences in virulence or pathogenicity can be a result of mutations in the genes encoding the envelope (E) protein glycosylation or the nonstructural (NS) protein [25].

The genome of WNV encodes a polyprotein that is processed into three structural proteins (a nucleocapsid protein, C; a precursor membrane glycoprotein, prM; and a glycosylated envelope protein, E) and seven non-structural (NS) proteins (NS1, NS2A, NS2B, NS3, NS4A, NS4B and NS5). Among them, the prM protein has a key role in the pathogenicity of WNV through particle secretion [26]. Moreover, the role of the E-protein is noted for pathogenicity as this protein plays a key role in membrane fusion between the virus and the host cell [27]. This happens via three different domains of E-protein including the Glycosaminoglycans (GAGs) binding domain, which can facilitate the initial attachment of viruses to its receptors on the cell surface [28,29]. In addition, the glycosylation site in the E protein is known as a virulence factor [17]. It has been noted that the introduction of a T249P amino acid substitution in the NS3 helicase is documented to increase virulence in birds and possibly other hosts [30]. In the NS4A, the PEPE motif is known for virion replication and release [31]. The NS-5 motif contains phenylalanine at position 653, which is associated with an increased resistance to interferon [32].

There is still no treatment or approved WNV vaccine for humans and our hope to prevent WNV outbreaks mainly relies on preventive strategies. The objective of a predictive oversight system is forecasting with a high degree of certainty the pathogenic potential of genomes of identified pathogens in comparison to the most closely related strains. Using sequence-based prediction; we aimed to study the mapping of the WNV genome, modeling of protein architecture and determination of virulence drivers at the genomic level. In addition, we aimed to investigate the origin of autochthonous WNV strains in Iran, their vector range and host-vector interactions.

## 2. Material and Methods

### 2.1. Study Area and Sampling

**Figure 1:**
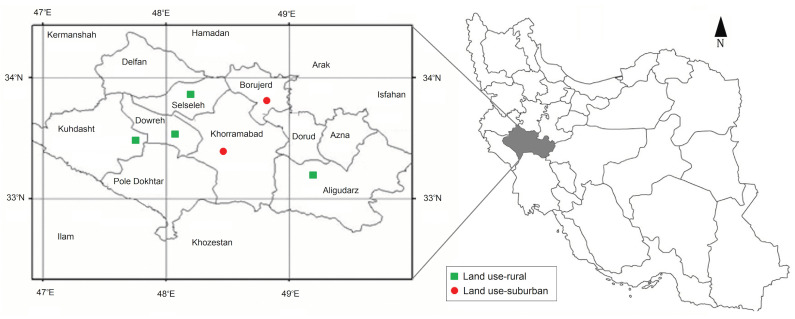
Trapping sites of the mosquitoes in this study, 2017–2018.

Mosquitoes were collected from six trapping sites including Khorramabad (suburban), Dowreh (rural), Kuhdasht (rural), Borujerd (suburban), Selseleh (rural) and Aligudarz (rural) in Lorestan province, Western Iran, from May to December 2017 and 2018 (Figure 1). In order to collect a wide range of mosquito species circulating in the area, we combined two different mosquito collection approaches; (i) BG-sentinel traps (BioGents, Regensburg, Germany) equipped with CO_2_ cylinder run continually (24 h per day) at each site during the trapping period. Sample collection nets were changed every two days. (ii) New Jersey light traps (John W. Hook Company, Gainesville, FL, USA) run from sundown to sunrise and mosquito samples were harvested by replacing a new collection net once a day. All traps were installed outdoor. Collected mosquitoes were stored in a −70 °C freezer and until further investigation [33]. At the end of each trapping season, samples were shipped in a cool box to the Medical Entomology Laboratory at Tehran University of Medical Sciences for analysis.

### 2.2. Morphological and Molecular Identification of Mosquitoes

Female mosquito samples were identified to species on chill boxes using discrimination keys of adult *Culicidae* mosquito species of Iran. Taxonomic keys were provided for the identification of the adult females and fourth-instar larvae of Iranian mosquitoes (Diptera: Culicidae). Newly recorded species, new characters, drawings illustrating characters used in the key [34]. Non blood-fed female mosquitoes of the same species and from the same location and collection period were pooled to a maximum of 250 individuals, whilst blood-fed mosquitoes were investigated individually [8]. From each pool or single mosquito sample, one leg was separated and transferred to a tube with 10 pieces of 1.0 mm zirconia beads (Kingda, Hunan, China) and 1 mL of cell culture medium (Sigma Aldrich, Munich, Germany) for homogenization. Then, DNA was extracted using the QIAamp Mini Kit (Qiagen, Hilden, Germany) to confirm the morphologically identified mosquito species and molecularly identify newly observed species. A DNA barcoding PCR reaction was then performed to amplify a 560 base-pair fragment of the Cytochrome oxidase I (COI), one of the most reliable markers for population genetic studies [35]. The amplified gene segment was sequenced and the mosquito samples were identified at the species level by BLAST. Further, we performed a multiplex real-time PCR using CQ11 microsatellite locus to discriminate between *Cpp.* biotypes for individual (blood-fed) *Cx. pipiens* specimens based on protocols described previously [36].

### 2.3. Blood Source Identification

Using specific primers designed previously [37], partial genomic DNA of the vertebrate hosts was amplified with the following temperature profile for each PCR reaction: incubation at 95 °C for 5 min, 40 cycles at 94 °C for 30 s, 57 °C for 30 s and 72 °C for 30 s and finally completed by incubation at 72 °C for 5 min. Through sequence blasting, the source of vertebrate blood in blood-fed mosquitoes was revealed [38].

### 2.4. Panel PCR for Arbovirus Screening

In order to screen both blood-fed and non-blood-fed female mosquitoes for arboviruses, RNA was extracted from homogenized specimens using the RNeasy Mini Kit (Qiagen, Germany). Then, a Pan RT-PCR approach was applied using Superscript IV (Invitrogen, Thermo Scientific, Germany) and corresponding specific primers for five main families of arboviruses, including *Togaviridae*, *Flaviviridae*, *Peribunyaviridae, Phenuiviridae and Rhabdoviridae* [39,40,41]. Thermal cycling conditions comprised of an initial RT step at 50 °C for 10 min, followed by 98 °C for 5 min and 45 cycles of denaturation at 98 °C for 10 s, annealing at 55 °C for 10 s and polymerization at 72 °C for 30 s. Samples positive for WNV were then further analyzed to obtain a full-length sequence with WNV-specific primers according to previously described protocols [8,42]. The PCR amplicons were subjected to Sanger sequencing and the sequences were deposited to GenBank under accession numbers MN238669 and MN238670.

### 2.5. Polyprotein Characterization

In order to predict pathogenic potential of obtained WNV sequences, known virulence motifs were determined in WNV genome and amino acid substitutions in these regions were investigated. To do this, multiple amino acid alignment was done using the MAFFT algorithm by Geneious version 11.1.2 software (Biomatters Ltd., Auckland, New Zealand). Furthermore, the homology models for WNV envelope protein was constructed using the Oligomeric modeling, which combined interface conservation, structural clustering and other template features to provide a quaternary structure quality estimate (QSQE) in the SWISS-MODEL workspace (http://swissmodel.expasy.org/workspace/). The secondary structure (α-helices and β-sheets) was extracted from the 3D structure using DSSP program by reading the position and energy between atoms [43].

### 2.6. Full Genome-Based Phylogenetic Tree Construction

In addition to sequences obtained in this study, a data set (WNV sequences from all lineages with diverse spatiotemporal profile) was extracted from GenBank (Table S1). The RNA sequences were subjected to alignment using MAFFT algorithm. In order to build the phylogenetic tree, the Tamura-Nei genetic distances model and Neighbour-Joining (NJ) method were selected with sorted topologies and 70% threshold. Analyses of the sequences were conducted using Geneious version 11.1.2 software. To assess the subclades of WNV lineage 2, splits network was made by EqualAngel method using the Splits Tree 4.0 software [44,45].

## 3. Results

### 3.1. Mosquito Population Dynamic

Of 5028 mosquito specimens collected, 4272 (85%) were within the *Culex (Cx)* genus: 2885 *Cx. pipiens* complex (57.4%), 1224 *Cx. theileri* (24.3%) and 163 *Cx. perexiguus* (3.2%). 756 (15%) were *Anopheles (An)*: 535 *An. sacharovi* (10.6%)*,* 83 *An. stephensi* (1.7%), 75 *An. superpictus* (1.5%), 32 *An. dthali* (0.6%), 28 *An. maculipennis* (0.6%) and 3 *An. fluviatilis* (0.1%) (Table 1). Moreover, DNA barcoding PCR confirmed the morphological identification of mosquitoes to species level in the subset samples (at least nine DNA extracts from mosquito specimens) covering all morphologically identified mosquito specimens (Table 1).

Seventy-six female mosquitoes were found to be blood-fed. They were from six species: *Cx. theileri* (*n* = 31; 40.8%), *An. sacharovi* (*n* = 4; 5.3%), *An. maculipennis* (*n* = 20; 26.3%), *Cpp.* biotype *pipiens* (*n* = 8; 10.5%), *Cx. perexiguus* (*n* = 11; 14.5%) and *An. superpictus* (*n* = 2; 2.6%). Their blood meal was derived from six different mammalian hosts, including humans and one bird species (21 mosquitoes fed on *Homo sapiens*, 18 on *Sus scrofa*, 16 on *Bos taurus*, 11 on *Canis lupus*, 3 on *Ovis aries* and 7 mosquitoes that fed on one single bird species (*Anas sparsa*) (Table 1).

### 3.2. Host-Vector Interaction

**Figure 2:**
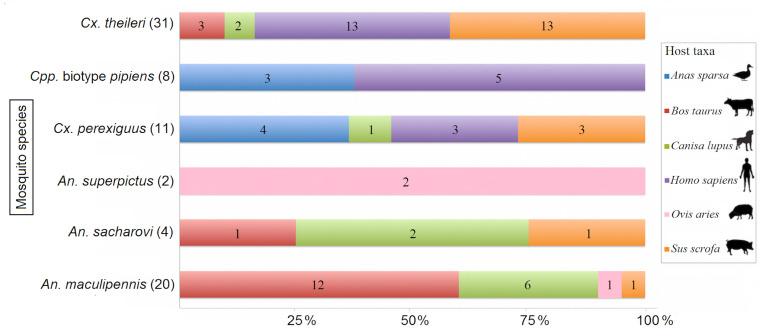
Host-feeding behavior of blood-fed mosquitoes from Western Iran.

Data analysis on mosquito species preference for a specific host revealed that from 8 blood-fed *Cpp.* biotype *pipiens*, 5 (62.5%) fed on *Homo sapiens,* while 3 (37.5%) fed on *Anas sparsa*. The host-feeding pattern for 31 blood-fed *Cx. theileri* is *Homo sapiens* (*n* = 13, 41.9%), *Sus scrofa* (*n* = 13; 41.9%), *Bos taurus* (*n* = 3; 9.7%) and *Canis lupus* (*n* = 2; 6.5%). Of 11 blood-fed *Cx. perexiguus*, 3 (27.3%) fed on *Homo sapiens,* 3 (27.3%) on *Sus scrofa,* 1 (9.1%) on *Canis lupus and* 4 (36.4%) on *Anas sparsa.* Of 4 blood-fed *An. sacharovi*, 1 (25%), 1 (25%) and 2 (50%) preferred *Sus scrofa, Bos taurus and Canis lupus* for feeding, respectively. All blood-fed *An. superpictus* fed on *Ovis aries.* The total number of blood-fed *An. maculipennis* was 20; their host preference was *Sus scrofa*, *Bos taurus*, *Canis lupus and Ovis aries,* with 1 (5%), 12 (60%), 6 (30%), 1 (5%), respectively (Figure 2).

The vertical axis shows the mosquito species and, in brackets, the number of individuals. The horizontal axis shows the blood meal identification in each mosquito species (percentage and number).

### 3.3. WNV Prevalence in Mosquito Specimens

In total, 42 mosquito pools (non-blood-fed) and 76 individual mosquitoes (blood-fed) were screened for five families of arboviruses. No arbovirus other than WNV was detected. WNV was identified in one mosquito pool containing 98 non-blood-fed *Cx. theileri* captured in August 2018 and one blood-fed *Cpp.* biotype *pipiens* captured in September 2017. The source of blood meal in the positive *Cpp.* biotype *pipiens* sample was a human. In order to determine whether the blood-fed *Cpp.* biotype *pipiens* was already infected with WNV or infection was from human blood meal, we tested the previously separated leg from this specimen, which proved WNV-positive.

### 3.4. Characterization of Full-Length Genome and Amino Acid Motifs

**Figure 3:**
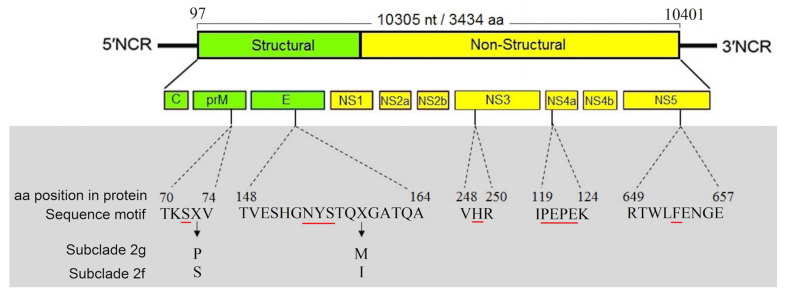
Schematic representation of the genomic structure of West Nile virus (WNV) with genes labeled (white area). Known virulence motifs identified in Iranian WNV specimens with amino acid substitutions in subclade 2g and subclade 2f (grey area). Key codons at each known virulence motifs are labeled by red underline.

The complete nucleotide sequences of the WNV strains obtained from *Cpp.* biotype *pipiens* (GenBank accession number: MN238669) and *Cx. theileri* (MN238670) are composed of 11,013 nt. One open reading frame consisting of 10,305 nt between nucleotide positions 97 and 10,401, coding for a 3,434 amino acid putative polyprotein precursor, was identified (Figure 3).

Five known virulence motifs are determined in WNV sequences obtained from Iran. At the amino acid motif (TKSS/PV) in the prM protein, we observed an evolutionary substitution at amino acid position 73 (serine in subclade 2f and proline in subclade 2g). Moreover, amino acid comparison showed that all Iranian WNV strains had the NYS motif at the glycosylation site (positions E-154 to E-156). Furthermore, WNV strains from Central African Republic (subclade 2f) have isoleucine at position E-159, while WNV strains from Eastern-Europe (subclade 2g) have methionine. The VHR, another known virulence motif, was also detected in Iranian WNV sequences, with a histidine residue at NS3-249. Any amino acid substitutions were not observed in the PEPE motif in the NS4a. The NS-5 motif contains phenylalanine at position 653 (Figure 3).

### 3.5. E-Protein Molecular Architecture

**Figure 4:**
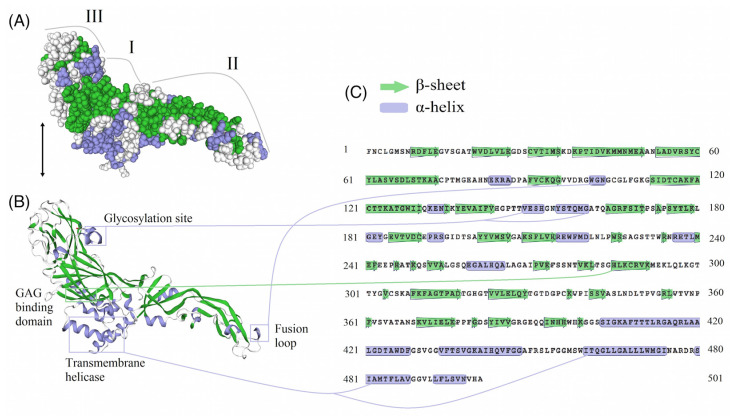
Predicted three-dimensional structure of an envelope glycoprotein monomer of the Iranian WNV sample. Surfaces of the E-protein model colored according to protein structure and three domains I, II and III are illustrated (**A**). The fusion loop, a two-helix transmembrane anchor, glycosylation site and GAG binding domain and correspondent amino acid sequence in E-protein are shown (**B**,**C**).

In order to assess the potential structural impact of changes in the E-protein, a three-dimensional structural homology model of E-protein was generated using the SWISS-MODEL workspace. Three distinct domains are present in the E-protein (Figure 4A). These domains are followed by a stem region, which contains two cationic amphipathic helices and ending in an anchor region with two transmembrane helices. Also, the Iranian WNV lineage 2 possesses a glycosylation site (Asn154), which is associated with virulent strains. The hydrophobic anchoring fusion loop (residues 98–109), which is present in the E-protein, has a key role in the penetration of the outer bilayer leaflet of the host cell membrane to initiate cell entry. The GAG binding domain (residues 285–291) typically contains positively charged amino acids and is present in the E-protein structure of WNV obtained from mosquitoes in Iran (Figure 4B,C).

### 3.6. Genetic Determinants of Virulence in Iranian WNV Strains

Although the virulence motifs were observed in highly neuroinvasive and medium/low neuroinvasive WNV strains, further amino acid substitution analysis in a subset of WNV strains with highest genetic similarity to Iranian WNV strains revealed subtle changes responsible for altering the pathogenicity of WNV strains. As a result, amino acid substitutions known as markers for neuroinvasive phenotype are observed in Iranian WNV strains. This amino acid substitution that alters attenuated strains to a virulent phenotype includes six mutations (Table 2).

At most of these positions, these amino acids vary between the different subclades. At position E-159, the less virulent strain (Madagascar/HM147823) has a valine residue, while in neuroinvasive strains an isoleucine in subclades 2f and methionine in subclade 2g are found. At position 338 in NS1, the attenuated Madagascar strain contains a leucine residue, whilst Iranian WNV strains and virulent lineage 2 strains have a threonine at this position. In protein NS2A, the amino acid substitution L126S was observed in virulent strains and Iranian WNV strains obtained in this study. At NS3-160, the less virulent strains (EF429199) and attenuated strain (HM147823) have alanine, while Iranian WNV strains and highly pathogenic strains have serine at this site. Moreover, NS3-S421 is a conserved residue in all lineages, except for attenuated lineage 2, which contain an asparagine. At position NS4B-20, leucine is replaced by proline in all neuroinvasive strains and Iranian WNV strains. The NS5-Y254 substitution to phenylalanine is a mutation found in virulent lineage 2 isolates as well as Iranian WNV strains. Furthermore, the sites PrM-73, E-71 and NS5-403 are subjected to adaptive evolution. Thus, mutations in these sites can result in increased WNV diversity (Table 2).

### 3.7. Evolutionary Tree of WNV Based on Complete Nucleotide Sequences

**Figure 5:**
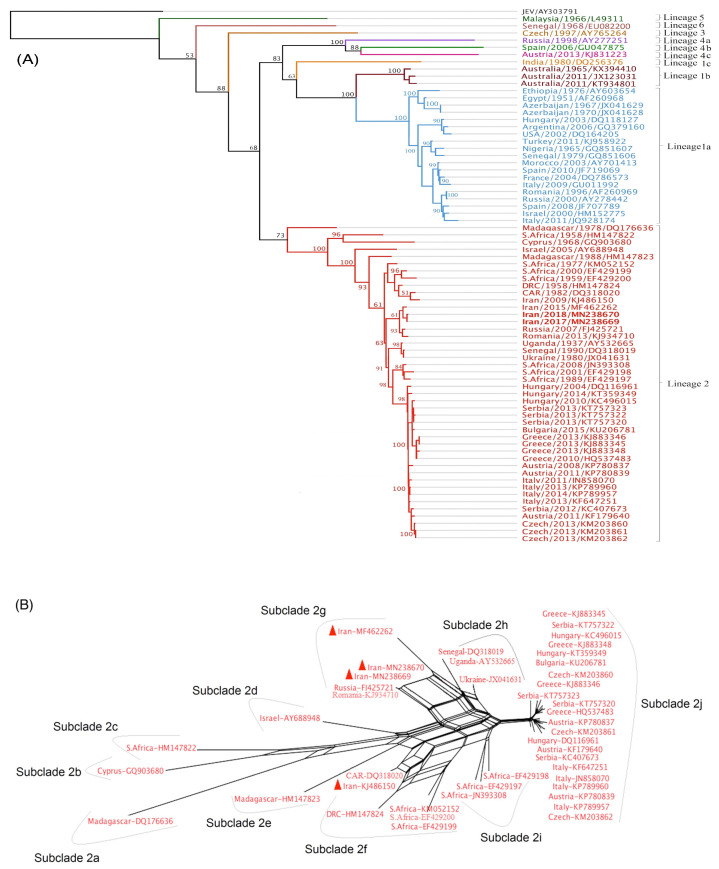
Phylogenetic analysis of WNV strains. The phylogenetic tree based on WNV full-length sequences was constructed with the Geneious software. The bootstrap values and number of bootstrap replications were greater than 50% and 1000, respectively. JEV: Japanese encephalitis virus (**A**). The neighbor-net network of lineage 2 was constructed using the Splits tree software. WNV sequences from Iran are marked with a red triangle (**B**). CAR stands for Central African Republic and DRC stands for Democratic Republic of Congo.

The phylogenetic analysis confirmed clustering of WNV sequences in six main lineages. The mosquito-derived Iranian WNV strains determined in this study fell within lineage 2 (Figure 5A). Further analysis with the Split network clearly revealed clustering of WNV lineage 2 into ten subclades designated as subclade 2a (Madagascar strain DQ176636), subclade 2b (Cyprus strain GQ903680), subclade 2c (South African strain HM147822), subclade 2d (Israeli strain AY688948), subclade 2e (Madagascar strain HM147823), subclade 2f (WNV strains of Central-South Africa), subclade 2g (Eastern-Europe), subclade 2h (Africa-Ukraine), subclade 2i (several South African strains) and subclade 2j encompassing Central European strains (Figure 5B).

The split tree showed that the Iranian mosquito-derived WNV sequences belong to subclade 2g together with other strains, for example, an Iranian mosquito-derived WNV strain from 2015 (MF462262), a strain of a Russian human patient in 2005 (FJ425721) and a WNV of a Romanian tick from 2013 (KJ934710). Interestingly, the previously isolated WNV strain from an Iranian encephalitis patient in 2009 clusters in subclade 2f, together with other WNV strains from the Central African Republic (DQ318020) and South Africa (EF429200) (Figure 5B).

## 4. Discussion

Emerging infectious diseases including WNV have a profound impact on global health. For instance, the WNV epidemics in urban areas in Romania in 1996 and Russia in 1999 affected many people, which developed WNV neuroinvasive disease and required hospitalization. Ongoing global environmental and socioeconomic changes may create favorable conditions for the emergence and transmission of vector-borne diseases in Iran via: (i) the importation of vectors and pathogens exotic to Iran through international movement such as aircraft, transoceanic shipping, trucking and so forth. [3,46]; and (ii) the re-emergence or resurgence of endemic vector-borne diseases, which may occur due to changes in the geographic range of vectors as a result of environmental and climate change [47].

**Figure 6:**
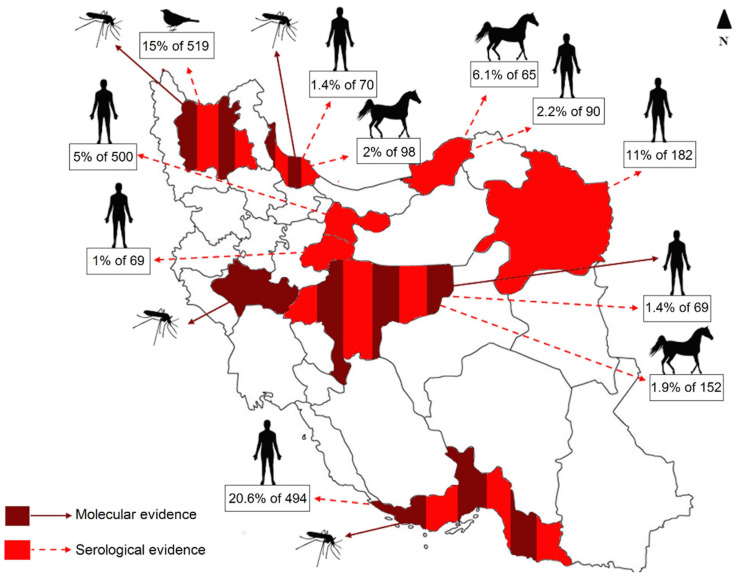
Serological (IgG) WNV evidence in human, horse and bird samples and molecular WNV evidence in humans and mosquitoes in Iran. The percentage of IgG positive samples to the number of samples tested is shown in boxes. White areas have not been investigated for WNV prevalence.

Current knowledge suggests the emergence of WNV in previously naive areas in Iran since in our previous studies in 2015 and 2016 all mosquito samples (n = 4211) collected in Lorestan province were negative for arboviruses [8,48]. However, a successive surveillance project for arboviruses revealed WNV in this province, where two mosquito specimens collected in 2017 and 2018 were positive for WNV. In addition to the previously reported WNV vectors in Iran (*Cx. pipiens* complex and *Ae. caspius*) [49], we report here *Cx. theileri* as a possible WNV vector. This is important, as *Cx. theileri* is one of the most common mosquito species in several regions of Iran, especially in the western regions. It is also noteworthy that we found WNV for the first time in *Cx. theileri* collected from the field, while the vector competence of *Cx. theileri* for WNV was already demonstrated in the laboratory [50]. Combining all previous serological evidences of WNV in human populations and animals as well as the molecular detection of WNV in mosquitoes and humans during several years, it seems that WNV has been circulating in Iran for some time, during which cases of illness have been reported sporadically in human patients (Figure 6) [4,42,51,52,53]. The current data demonstrate the evidence of WNV in 9 out of 31 provinces of Iran but the exact burden of West Nile disease is still not fully understood in Iran yet and it can be assumed that there are more undetected human cases.

Molecular techniques have a critical role in the expansion of our knowledge on blood-feeding patterns of hematophagous arthropods [54,55]. In a previous study, the blood-feeding behavior of female mosquitoes in Iran was investigated, however, none of the blood-fed mosquitoes were positive for arboviruses [38]. The current data show that mosquito species fed on six different vertebrate species (mammals and one bird species). Of note, *Cx. theileri*, *Cx. perexiguus* and *An. maculipennis* fed on four host taxa. Other mosquito species including *An. sacharovi, Cpp.* biotype *pipiens and An. superpictus* fed on three, two and one host taxa, respectively. Combining the findings of Fritz et al. that *Cpp.* biotype *pipiens* mainly fed on birds [56] and our current findings with WNV detection in a *Cpp.* biotype *pipiens* fed with human blood emphasizes the crucial role of this mosquito species as bridge vector for transmission of WNV, which circulates between birds but occasionally infects humans. Since host availability is a major driver of host selection by mosquitoes, it can be assumed that the risk of accruing WNV infection in natural sites, where birds act as reservoirs, human and horses as dead end hosts and mosquitoes as vectors (specifically *Cx. pipiens*), could be higher than in urban areas [57].

This study is the first attempt to obtain and characterize a full-length WNV sequence in Iran. Phylogeny of the complete WNV genome obtained in this study and all previous Iranian WNV sequences obtained from a patient with encephalitis in 2009 and mosquito specimens collected in different years confirmed that circulating WNV strains in Iran belong to lineage 2 [58]. When splitting WNV strains into subclades, we could demonstrate co-circulation of two subclades with different origins in Iran: (i) in 2009, a subclade 2f strain was detected in a human patient from central Iran and (ii), between 2015 and 2018 subclade 2g (East-Europe) strains were detected in several mosquito species from northwest and western regions of Iran. These findings suggest two separate WNV introduction events into Iran.

Combining the data of the WNV evolutionary tree obtained in this study and the migratory bird flyway routes in the world [59], it is speculated that the first WNV introduction event occurred earlier from Central/East Africa to Central/South regions of Iran potentially through migratory birds that fly the East Africa-West Asia Flyway, while the second introduction happened later from Eastern Europe (probably Russia or Romania) to northwestern/western regions of Iran potentially through migratory birds in the Black Sea-Mediterranean Flyway. Moreover, comparison between WNV strains from Turkey and Northwestern Iran, where two countries share borders, suggest WNV introduction events to the Middle East with the same origin (Central Africa) are independent from each other and likely depend on the migratory bird flyways. This can be explained as an Iranian WNV strain (KJ486150) isolated from a patient shows similarity to a WNV strain from the Central African Republic (CAR), both grouping in lineage 2, whereas a WNV strain obtained from a horse in Turkey showed close genetic relationship with strain ArB310/67 from CAR, both grouped in lineage 1 [60].

The WNV sequences obtained in the current study had Serine at position prM-72, third amino acid at the motif TKSS/PV. The 72th amino acid position of the prM protein has shown to play a role in enhancing the virulence and particle secretion in WNV (Figure 3; red underline). Moreover, we observed an evolutionary amino acid substitution at position 73. In the 154–156th positions of the E-protein, we could identify the NYS motif (N-glycosylation motif) in Iranian WNV strains. According to previous studies, this site is present in neuroinvasive lineage 2 strains. Any amino acid substitutions or deletion of NYS can deteriorate virulence phenotype [61]. At codon position 249th, several variations have been reported in previous studies. At this site, proline is known to increase viremia and virulence in birds and probably in other hosts [62]. However, WNV sequences from Iran lack proline at NS3-249 and have a histidine residue instead (Figure 3; red underline). In addition, previous studies demonstrated that mutations in the conserved PEPE motif of the NS4a protein can attenuate or impair virion replication rates and protein production in Vero cells and are associated with low virulence strains in vivo. However, PEPE motif in our WNV sequences had no amino acid substitutions. The WNV strains obtained in the current study, contained phenylalanine at NS5-653, which is a virulence phenotype due to an increased resistance to interferon (Figure 3; red underline).

In order to identify whether mutations that have been shown to influence WNV virulence and replication were present in Iranian WNV strains isolated from a human patient in 2009 and mosquitoes in 2017–2018, Iranian strains were compared with lineage 2 strains with known high and low pathogenicity, respectively (Table 2). The analysis revealed the existence of several amino acid substitutions recognized as altering an attenuated strain to a virulent phenotype in Iranian WNV strains including E-V159I/M, NS1-L338T, NS2A-L126S, NS3-N421S, NS4B-L20P and NS5-Y254F [63]. Furthermore, several sites were found to have adaptive evolution roles in subclade 2f and subclade 2g, including prM-73, E-71 and NS5-403. Thus, mutations in these sites can result in WNV strain diversity with different levels of virulence [30]. The NS5-Y254 substitution to phenylalanine is a mutation found in virulent lineage 2 isolates. This position is located in the methyltransferase (MTase) domain of NS5 proximal to the RNA-binding site [64]. Mutations in the NS proteins encoding viral replication and protein cleavage mechanisms are the most likely determinants of differences in pathogenicity. The NS3 and NS4B proteins of less and highly neuroinvasive strains from Africa and Europe manifested hydrophobic and hydrophilic changes, which may have potential structural changes that affect function and, by implication, virulence [65].

In addition to the evaluation of virulence motifs and mutations, the E-protein structure was analyzed to gain a deeper insight into epitopes in the hot spot domains known to facilitate virus invasion and tropism in neuronal cells. As a result, known neuroinvasive epitopes were observed in α-helices glycosylation site, GAG binding domain and fusion loop, which are responsible for virus tropism and attachment to the cell surface [27]. This implies that Iranian WNV strains exhibit known virulence factors common to more neuroinvasive WNV variants [66].

## 5. Conclusions

Considering data from the current study and previous studies, autochthonous WNV circulation in Iran has been proven. Thus, creating awareness for this disease among clinicians is of the utmost importance. Also, hospital laboratories should establish WNV assays and include WNV in their neurological investigation panel. The results of this study will have practical implications for the improvement of public health policies by better understanding WNV transmission modes in Iran. It is hoped that important insights will be gained in the circulation, transmission and evolution of WNV, which will provide both short and long-term health and economic benefits to the Iranian population.

## Figures and Tables

**Table 1:** Total number of mosquito species sampled and origin of the blood meals identified in different mosquito species during 2017–2018, Iran.

Mosquito Species	No. of Mosquitoes/Species and (%) of Total	No. of Blood-Fed Mosquitoes/Species and (%) of Total	No. of Blood-Fed Mosquitoes/Host and (%) of Total					
			*Homo Sapiens*	*Sus* *Scrofa*	*Bos Taurus*	*Canis Lupus*	*Ovis* *Aries*	*Anas Sparsa*
***Cx. pipiens* Co.**	2885 (57.4)	8 (10.5)	5 (23.8)	0 (0)	0 (0)	0 (0)	0 (0)	3 (42.9)
***Cx. theileri***	1224 (24.3)	31 (40.8)	13 (61.9)	13 (72.2)	3 (18.8)	2 (18.2)	0 (0)	0 (0)
***Cx. perexiguus***	163 (3.2)	11 (14.5)	3 (14.3)	3 (16.7)	0 (0)	1 (9.1)	0 (0)	4 (57.1)
***An. sacharovi***	535 (10.6)	4 (5.3)	0 (0)	1 (5.6)	1 (6.3)	2 (18.2)	0 (0)	0 (0)
***An. stephensi***	83 (1.7)	0 (0)	0 (0)	0 (0)	0 (0)	0 (0)	0 (0)	0 (0)
***An. superpictus***	75 (1.5)	2 (2.6)	0 (0)	0 (0)	0 (0)	0 (0)	2 (66.7)	0 (0)
***An. dthali***	32 (0.6)	0 (0)	0 (0)	0 (0)	0 (0)	0 (0)	0 (0)	0 (0)
***An. maculipennis***	28 (0.6)	20 (26.3)	0 (0)	1 (5.6)	12 (75)	6 (54.5)	1 (33.3)	0 (0)
***An. fluviatilis***	3 (0.1)	0 (0)	0 (0)	0 (0)	0 (0)	0 (0)	0 (0)	0 (0)
**Total**	**5028 (100)**	**76 (100)**	**21**	**18**	**16**	**11**	**3**	**7**

**Table 2:** Amino acid substitutions observed in two WNV strains obtained in this study compared with highly or less pathogenic variants in subclade 2f and subclade 2g and a strain from subclade 2e (Madagascar/HM147823) as a less pathogenic strain. Amino acid substitutions known as markers for neuroinvasiveness and less virulent phenotypes are shown in red and green, respectively and mutation sites known as adaptive evolution sites are shown in grey.

Gene		Strain/Subclade/Pathogenicity	Iran (MN238670)/2g	Iran (MN238669)/2g	Russia (FJ425721)/2g/high	SA (EF429200) /2f/high	SA (EF429199)/2f/less	CAR (DQ318020)/2f/high	MAD (HM147823)/2e/attenuated
	a.a Position								
C	-		-	-	-	-	-	-	-
prM	73		P	-	-	S	S	S	S
	91		-	-	-	-	-	K	-
	105		A	-	-	-	V	-	-
	221		A	-	-	G	-	-	-
E	66		S	-	-	N	-	-	-
	70		T	-	-	P	-	-	-
	71		K	-	-	R	R	R	-
	154		N	-	-	-	-	D	-
	159		M	-	-	I	I	I	V
	250		Q	-	-	-	-	R	-
	312		A	-	-	-	V	-	-
NS1	88		V	-	-	-	I	I	-
	98		K	-	-	-	-	R	-
	290		S	-	-	-	R	-	-
	338		T	-	-	-	-	-	L
	401		I	-	V	-	-	-	-
	442		L	-	M	-	-	-	-
NS2A	126		S	-	-	-	-	-	L
	190		K	-	-	R	-	-	-
	259		S	-	-	T	-	-	-
NS2B	-		-	-	-	-	-	-	-
NS3	160		S	-	-	-	A	-	A
	298		R	-	-	-	G	-	-
	333		I	-	-	-	V	-	-
	421		S	-	-	-	-	-	N
	572		T	-	-	M	-	-	-
	597		A	-	-	V	-	-	-
NS4A	98		E	-	-	-	-	D	-
	100		P	-	S	-	-	-	-
NS4B	20		P	-	-	-	-	-	L
	117		A	-	-	V	-	-	-
	274		H	Y	Y	-	-	-	-
NS5	42		H	-	-	-	Q	-	-
	117		K	-	-	-	R	-	-
	254		F	-	-	-	-	-	Y
	278		K	-	-	-	R	-	-
	281		N	-	-	-	S	-	-
	370		E	-	A	-	-	-	-
	403		R	-	-	G	G	G	-
	528		P	-	-	-	G	-	S
	816		A	-	-	S	-	S	-
	837		K	-	-	R	-	R	-

## Supplementary Materials

Supplementary materials can be found at https://www.mdpi.com/1999-4915/12/12/1449/s1.
