# Supplementary material for: Autochthonous Transmission of West Nile Virus by a New Vector in Iran, Vector-Host Interaction Modeling and Virulence Gene Determinants
Source: Viruses. 2020 Dec 16;12(12):1449. doi: 10.3390/v12121449 (PMC7766443; doi:10.3390/v12121449)
Supplement: Supplementary file 1 [file viruses-12-01449-s001.pdf]

Table S1. West Nile virus (WNV) sequences from GenBank used for computational analysis, with associated countries of origin, isolation year, host, and GenBank accession number.

Country	Year	Strain	Lineage	Host	Accession No.
**Argentina**	2006	ArEq001	1a	Horse	GQ379160
**Australia**	2011	K74015	1b	Mosquito	KT934801
**Australia**	1965	Kunjin	1b	Bird	KX394410
**Australia**	2011	V11-07	1b	Horse	JX123031
**Austria**	2008	Austria-2008	2	Bird	KF179640
**Austria**	2011	Berliner	2	Bird	KP780839
**Austria**	2008	Sepperl	2	Bird	KP780837
**Austria**	2013	Uu-LN-AT	4c	Mosquito	KJ831223
**Azerbaijan**	1967	LEIV-1628Az	1a	Bird	JX041629
**Azerbaijan**	1970	LEIV-72Az	1a	Tick	JX041628
**Bulgaria**	2015	Sofia	2	Human	KU206781
**CAR***	1982	ArB3573	2	Tick	DQ318020
**Cyprus**	1968	Q3574	2	Not available	GQ903680
**Czech Rep**	2013	CZ13-104	2	Mosquito	KM203860
**Czech Rep**	2013	Cz13-329	2	Mosquito	KM203861
**Czech Rep**	2013	Cz13-479	2	Mosquito	KM203862
**Czech Rep**	1997	Rabensburg	3	Mosquito	AY765264
**DRC****	1958	DRC	2	Not available	HM147824
**Egypt**	1951	Eg101	1a	Human	AF260968
**Ethiopia**	1976	EthAn4766	1a	Not available	AY603654
**France**	2004	407-04	1a	Bird	DQ786573
**Greece**	2010	Nea Santa	2	Mosquito	HQ537483
**Greece**	2013	Kavala2	2	Human	KJ883348
**Greece**	2013	Thessaloniki4	2	Human	KJ883346
**Greece**	2013	Xanthi3	2	Mosquito	KJ883345
**Hungary**	2010	578-10	2	Horse	KC496015
**Hungary**	2003	Goose-Hungary-03	1a	Bird	DQ118127
**Hungary**	2004	Goshawk	2	Bird	DQ116961
**Hungary**	2014	hun	2	Human	KT359349
**India**	1980	804994	1c	Human	DQ256376
**Iran**	2009	Iran-WNV	2	Human	KJ486150
**Iran**	2015	SepidRiver	2	Mosquito	MF462262
**Israel**	2005	Sarafend	2	Not available	AY688948
**Israel**	2000	ISR00	2	Human	HM152775
**Italy**	2014	Cremona4	2	Human	KP789957
**Italy**	2009	Ita09	1a	Human	GU011992
**Italy**	2011	Livenza	1a	Human	JQ928174
**Italy**	2013	Mantova	2	Human	KP789960
**Italy**	2013	Padova34	2	Human	KF647251
**Italy**	2011	AN-2	2	Human	JN858070
**Madagascar**	1978	AnMg798	2	Bird	DQ176636
**Madagascar**	1988	Madagascar	1a	Not available	HM147823
**Malaysia**	1966	Kunjin- Sarawak	5	Mosquito	L49311
**Morocco**	2003	04.05	1a	Horse	AY701413
**Nigeria**	1965	IBAN7019	1a	Not available	GQ851607
**Romania**	2013	Hyalomma	2	Tick	KJ934710
**Romania**	1996	RO97-50	1a	Mosquito	AF260969
**Russia**	1998	LEIV-Krnd88-190	4a	Tick	AY277251
**Russia**	2000	LEIV-Vlg00-27924	1a	Human	AY278442
**Russia**	2007	Reb-VLG	2	Human	FJ425721
**S.Africa**	1977	349-77	1a	Horse	KM052152
**S.Africa**	1958	H442/high	2	Human	EF429200
**S.Africa**	2008	HS101	2	Horse	JN393308
**S.Africa**	2000	SA381/less	2	Human	EF429199
**S.Africa**	2001	SA93/01/high	2	Human	EF429198
**S.Africa**	1958	SA	2	Not available	HM147822
**S.Africa**	1989	SA	2	Human	EF429197
**Senegal**	1979	ArD27875	1a	Not available	GQ851606
**Senegal**	1968	Koutango-DakAaD	6	Mosquito	L48980

**Senegal**	1990	ArD76104	2	Mosquito	DQ318019
**Serbia**	2012	Sad12	2	Bird	KC407673
**Serbia**	2013	Vojvodina	2	Mosquito	KT757320
**Serbia**	2013	Vojvodina	2	Mosquito	KT757322
**Serbia**	2013	Vojvodina	2	Mosquito	KT757323
**Spain**	2010	H-1b	1a	Horse	JF719069
**Spain**	2006	HU2925	4b	Mosquito	GU047875
**Spain**	2008	HU6365-08	1a	Mosquito	JF707789
**Turkey**	2011	T2	1a	Horse	KJ958922
**Uganda**	1937	B956	2	Human	AY532665
**Ukraine**	1980	LEIV-3266Ukr	2	Bird	JX041631
**USA**	2002	TX.2002	1a	Human	DQ164205

* CAR: Central African Republic; ** DRC: Democratic Republic of Congo.
